# Exploring Borate-Modified Calcium Phosphate Ceramics: Antimicrobial Potential and Cytocompatibility Assessment

**DOI:** 10.3390/nano14060495

**Published:** 2024-03-09

**Authors:** Inna V. Fadeeva, Katia Barbaro, Annalisa Altigeri, Anna A. Forysenkova, Marat R. Gafurov, Georgy V. Mamin, Alexander V. Knot’ko, Viktoriya G. Yankova, Anna A. Zhukova, Fabrizio Russo, Julietta V. Rau

**Affiliations:** 1 A.A. Baikov Institute of Metallurgy and Material Science, Russian Academy of Sciences, Leninsky Prospect 49, 119334 Moscow, Russia; fadeeva_inna@mail.ru (I.V.F.); aforysenkova@gmail.com (A.A.F.); 2 Istituto Zooprofilattico Sperimentale Lazio e Toscana “M. Aleandri”, Via Appia Nuova 1411, 00178 Rome, Italy; katia.barbaro@izslt.it (K.B.); annalisa.altigeri@izslt.it (A.A.); 3 Institute of Physics, Kazan Federal University, Kremlyovskaya St. 18, 420008 Kazan, Russia; marat.gafurov@kpfu.ru (M.R.G.); george.mamin@kpfu.ru (G.V.M.); 4 Chemistry Department, Lomonosov Moscow State University, Leninskie Gory 1, 119991 Moscow, Russia; alknt@mail.ru; 5 Department of Analytical, Physical and Colloid Chemistry, I.M. Sechenov First Moscow State Medical University, Trubetskaya 8, Build. 2, 119048 Moscow, Russia; yankova_v_g@staff.sechenov.ru (V.G.Y.); zhukova_a_a@staff.sechenov.ru (A.A.Z.); 6 Laboratory of Regenerative Orthopaedics, Research Unit of Orthopaedic, Department of Medicine and Surgery, Università Campus Bio-Medico di Roma, Via Alvaro del Portillo 21, 00128 Rome, Italy; fabrizio.russo@policlinicocampus.it; 7 Operative Research Unit of Orthopaedics, Fondazione Policlinico Universitario Campus Bio-Medico, Via Alvaro del Portillo 200, 00128 Rome, Italy; 8 Istituto di Struttura della Materia, Consiglio Nazionale delle Ricerche (ISM-CNR), Via del Fosso del Cavaliere 100, 00133 Rome, Italy

**Keywords:** boron, borate, tricalcium phosphate, borate doped tricalcium phosphate, ceramics, antibacterial, bactericidal, osteogenic differentiation

## Abstract

Addressing periprosthetic infections, which present significant healing challenges that often require revision surgeries, necessitates the development of novel antibacterial materials and implants. Current research focuses on creating materials that hinder bacterial adhesion, colonization, and proliferation in surrounding tissues. Boron (B)-containing compounds are known for their antibacterial properties and potential in bone metabolism for regenerative medicine. In this study, we synthesized B-containing tricalcium phosphate (0.3B-TCP) with 1.1 wt.% B content via precipitation from aqueous solutions and sintering at 1100 °C. X-ray diffraction confirmed the ceramic’s primary crystalline phase as β-TCP, with B evenly distributed according to energy-dispersive spectroscopy data. Electron paramagnetic resonance (EPR) data verified stable paramagnetic borate anions, indicating successful BO_3_^3−^ substitution for phosphate groups. The microstructural properties of 0.3B-TCP ceramic were assessed before and after soaking in a saline solution. Its bending strength was approximately 30 MPa, and its porosity was about 33%. 0.3B-TCP ceramic demonstrated significant antimicrobial efficacy against various bacterial strains and a fungus. Cytotoxicity evaluation using equine adipose tissue-derived mesenchymal stem cells and osteogenic differentiation assessment were conducted. The combination of antibacterial efficacy and good cytocompatibility suggests 0.3B-TCP ceramic as a promising bone substitute material.

## 1. Introduction

The interdisciplinary field of bone tissue engineering focuses on the development of biocompatible and multifunctional materials and devices that facilitate the regeneration of bone tissue [1]. Through the utilization of calcium phosphates (CPs), bioactive ceramics can be produced as bulk biomaterials, coatings for bone implants, or reinforced composite frameworks. Owing to its remarkable osteoconductive capability, calcium phosphates have been extensively employed in orthopedics since the mid-twentieth century [2]. A significant challenge complicating the healing process and often necessitating revision surgeries is periprosthetic infection. Estimates indicate that up to 2.5% of primary hip and knee arthroplasties and up to 20% of revision arthroplasties face complications due to periprosthetic joint infection (PJI) [3]. The socioeconomic impact of periprosthetic infections is significant and multifaceted. Indeed, they represent a considerable challenge, often leading to prolonged hospital lengths of stay, increased healthcare costs, and decreased quality of life for patients. Revision surgeries and potential long-term complications further exacerbate the financial burden on healthcare systems.

Some authors argue that these values are not only underestimated, but also on the rise [4]. *Staphylococcus aureus* (*S. aureus*), particularly methicillin-resistant, is one of the major contributors to both surgical site infections (SSIs) and periprosthetic joint infections (PJIs) [5]. While Gram-positive bacteria, mainly *S. aureus* and coagulase-negative staphylococci, account for the majority of PJIs, Gram-negative bacilli, such as *Pseudomonas aeruginosa* (*P. aeruginosa*), contribute to about 23%, especially in early-onset infections [6]. These germs can form resilient biofilms, hindering attacks by the immune system and reducing antibiotic diffusion, thus compromising efficacy [7,8,9]. Deep infections often necessitate implant removal, leading to increased morbidity and mortality, coupled with substantial therapy costs [7]. In this scenario, research should be focused on the development of effective antibacterial materials and biomedical implants based on those that prevent bacterial adhesion, colonization, and proliferation in the surrounding tissues.

A promising approach involves incorporating ions with antibacterial properties, such as zinc, copper, gadolinium, manganese, etc., into bioresorbable CP matrices. This not only introduces antibacterial activity but also enhances the rate of bioresorption compared to matrices without ion doping [10,11,12,13].

Boron (B)-containing substances are also known to exhibit antibacterial activity. The antibacterial activity of various boron compounds against biofilm pathogens was investigated in ref. [14,15]. In particular, boron cluster compounds possess the potential to emerge as novel chemical leads in antimicrobial therapy due to their derivatives exhibiting promising antimicrobial activity and low susceptibility to both phenotypic and genetic mechanisms of pathogen resistance [16]. These inorganic compounds have chemical compositions and structures that differ significantly from those of organic compounds, forming a diverse family of molecules. Characterized by 3-dimensional, aromatic structures, like (B–B–B and B–B–C), with 3-center-2-electron (3c2e) bonds, boron cluster compounds are not naturally occurring in living organisms. This absence results in a lack of enzymatic systems capable of metabolizing these compounds, contributing to their high biological stability. Furthermore, boron cluster compounds interact with components of biological systems, such as proteins and lipid membranes, through mechanisms distinct from those of organic compounds. As a consequence, derivatives of boron cluster compounds demonstrate equal efficacy against both standard and multi-drug resistant strains of pathogens. Additionally, they exhibit antibiofilm activity and are less prone to induce drug resistance [16].

As an example, boron-cluster penicillin analogs were prepared and investigated in [17]. The most potent conjugate exhibited the same activity as penicillin G against *S. aureus*.

On the other hand, boron has been identified as playing a significant role in bone metabolism and holding promise for regenerative medicine [18,19,20,21,22,23]. The mechanism of boron’s effect on the survival and proliferation of osteoblastic MC3T3-E1 cells, as well as the mRNA expression of mineralized tissue-associated proteins, was investigated in [24]. The study revealed that B acts as a dose-dependent regulator of osteoblast cells in over a short period (24 h). However, in the long term, there was no statistically significant difference in different boron concentrations compared to the control group.

Existing literature on boron calcium phosphates primarily discusses biphasic ceramics composed of a mixture of boron-doped hydroxyapatite/β-TCP obtained through the wet precipitation method [25,26]. Nevertheless, studies on boron-substituted hydroxyapatite as the main phase, obtained through both solid-state synthesis [27,28] and wet precipitation [29], are also available.

It is important to note that, to the best of our knowledge, investigations into the antibacterial and cytotoxic effects of boron-substituted tricalcium phosphate ceramics are lacking in the existing literature.

Therefore, the objective of this study was to incorporate boron into β-tricalcium phosphate (TCP), a well-known resorbable bioceramic material. Precipitation from an aqueous solution was utilized as the synthesis method for B-TCP. Its physicochemical characterization was conducted using X-ray diffraction (XRD), electron paramagnetic resonance (EPR) spectroscopy, and scanning electron microscopy (SEM), coupled with energy-dispersive X-ray (EDX). The porosity, mechanical properties, and solubility of the B-TCP ceramic in a saline solution were investigated.

The main focus of this study was to assess, for the first time, the antibacterial properties of the prepared ceramics against two Gram-positive strains (*S. aureus*, *Enterococcus faecalis* (*E. faecalis*)), two Gram-negative strains (*P. aeruginosa*, *Escherichia coli* (*E. coli*)), and the fungus *Candida albicans* (*C. albicans*)). Equine adipose tissue-derived mesenchymal stem cells (aMSCs) were employed to assess the cytotoxicity of the prepared materials. The cells’ viability was evaluated using the MTT assay, and the osteogenic differentiation was investigated.

## 2. Materials and Methods

### 2.1. Synthesis Procedure

β-tricalcium phosphate (Ca_3_(PO_4_)_2_, TCP) and boron-containing β-tricalcium phosphate (0.3B-TCP) were synthesized through precipitation from aqueous solutions of calcium nitrate and ammonium dihydrogen phosphate with a Ca/(P + B) ratio of 1.5, according to the following reactions:

3Ca(NO_3_)_2_ + 2(NH_4_)_2_HPO_4_ → Ca_3_(PO_4_)_2_ + 4NH_4_NO_3_ + 2HNO_3_ (1)

3Ca(NO_3_)_2_ + 1.99(NH_4_)_2_HPO_4_ + 0.3H_3_BO_3_ → Ca_3_(PO_4_)_1.99_(BO_2_)_0.3_ + 4NH_4_NO_3_ + 2HNO_3_ (2)

Chemical analysis-grade reagents (Labtech, Moscow, Russia) were employed for the syntheses. Solutions of calcium nitrate, ammonium dihydrogen phosphate, and boric acid were prepared at a concentration of 0.5 mol/L. A mixture of 398 mL of ammonium phosphate solution and 1.86 g of boric acid in 100 mL of distilled water was slowly added to 600 mL of calcium nitrate solution while stirring. The acidity of the reaction mixture was maintained in the pH range of 6.5–6.8 by adding a 25% aqueous solution of ammonia. The resulting precipitate was separated after 30 min by filtration using a Buchner funnel, dried at 105 °C, and subjected to calcination at 900 °C for 1 h to convert amorphous calcium phosphate to β-TCP. Subsequently, the materials were ground with corundum balls in a planetary mill in an isopropyl alcohol medium.

### 2.2. Physicochemical Characterization

XRD analysis was performed using a Rigaku D/MAX-2500V/PC diffractometer (Tokyo, Japan) with CuKα radiation (λ = 0.15418 nm).

EPR measurements were carried out using the X-band (with a microwave frequency of 9.5 GHz) Bruker Elexsys E580 spectrometer (Karlsruhe, Germany) in both continuous (CW) and pulse modes. Stable paramagnetic centers were formed under X-ray irradiation of synthesized powders using the URS-55 source (St. Petersburg, Russia, W-anticathode) at room temperature for 30 min with an estimated dose of 5 kGy [30]. Analysis (simulation) of the EPR spectra was carried out using Matlab 2018b with the Easyspin software package, version 5.2.0 [31].

To produce ceramic samples, TCP and B-TCP powders were pressed into 4 × 40 mm steel molds using a force of 800 kg. Subsequently, the resulting samples were sintered in a chamber furnace with silite heaters at a temperature of 1100 °C for 2 h.

The solubility of the ceramics was investigated at a temperature of 37 °C in a saline solution (0.9% sodium chloride solution) containing a TRIS buffer at pH 7.4. The ceramic/saline ratio was 0.5 g/100 mL. The concentration of calcium ions was measured using an atomic absorption spectrophotometer iCE 3500 (Thermo Fisher Scientific, Waltham, MA, USA).

Ceramic samples for mechanical tests were molded by uniaxial pressing in steel molds at a specific pressing pressure of 100 MPa, followed by sintering in a furnace with silite heaters at a temperature of 1100 °C for 1 h. The resulting ceramic samples had rectangular shapes with dimensions of 3 × 3 × 30 mm. The bending strength of the ceramics was examined using an Instron 5581 instrument (Instron Inc., Norwood, MA, USA).

The microstructure and elemental distribution of the ceramics were analyzed using a Tescan Vega II (Brno, Czech Republic) scanning electron microscope equipped with an energy-dispersive X-ray detector.

The porosity of the ceramics was determined by hydrostatic weighing following the method described in [32].

### 2.3. Microbiology Tests

The antimicrobial activity of pure β-TCP and boron-doped 0.3B-TCP ceramics was studied using *S. aureus*, *E. coli*, *E. faecalis*, *P. aeruginosa*, and *C. albicans*. The growth of microorganisms was assessed at an optical density (OD) of 600 nm wavelength by means of a Biophotometer D30 (Eppendorf, Hamburg, Germany).

Both materials were autoclaved at 121 °C for 20 min. For each material, 0.02 g was weighed and dissolved in 50 mL of Brain Heart Infusion (BHI, DIFCO, Sparks, NV, USA). In each test, 1.9 mL was taken, and 0.1 mL of a suspension of microorganisms diluted in BHI was added (OD600 = 0.01). The tests were conducted in triplicate.

The bacteria (*S. aureus*, *E. coli*, *P. aeruginosa*, and *E. faecalis*) were cultured at 37 °C, and the fungus (*C. albicans*) was cultured at 28 °C for 24 h.

### 2.4. Isolation of Mesenchymal Stromal Cells

Mesenchymal stromal cells (aMSCs) were isolated from the adipose tissue of a slaughtered male horse, approximately two years of age. Adipose tissue was cut into small pieces and digested at 37 °C with 0.1% collagenase 1A (Sigma-Aldrich, Edinburgh, UK) for 1 h under stirring. Following enzymatic digestion, the mixture was centrifuged at 800× *g* for 10 min, and the pellet was re-suspended in DMEM growth medium (Gibco, UK) with 10% FBS (Fetal Bovine Serum, Gibco, UK). The obtained cell suspension was seeded into flasks and incubated at 37 °C with 5% CO_2_. The growth medium was refreshed every 2–3 days with fresh medium.

### 2.5. MTT Test

The MTT test was employed to assess the toxicity of TCP and 0.3B-TCP. The materials were sterilized in an autoclave at 121 °C for 20 min. The adipose tissue-derived mesenchymal stromal cells (aMSCs) at the third passage, approximately at 80% confluence of the cell monolayer, were collected and distributed into 24-well flasks at a concentration of 50,000 cells/mL.

After 24 h, at 37 °C in a 5% CO_2_ environment, the medium was replaced with growth medium with 0.2 μg/mL of TCP and 0.2 μg/ mL of 0.3B-TCP. Only 1 mL of growth medium was added to the positive control cells. Each test was repeated three times. Following 24 h of incubation at 37 °C in a 5% CO_2_ environment, the supernatant of all wells was replaced with 1 mL of MTT (Sigma-Aldrich, UK) at a concentration of 0.5 mg/mL in DMEM. After incubation at 37 °C for 3 h, the MTT solution was replaced with isopropanol (Sigma-Aldrich, UK) and incubated for 30 min at room temperature. The solubilized formazan was measured at an OD of 600 nm using a biophotometer (Eppendorf, Hamburg, Germany).

### 2.6. Osteogenic Differentiation

For osteogenic differentiation, 50,000 cells/mL of aMSCs at the third passage were utilized. Cells were harvested in growth medium and seeded into 6-well flasks, followed by incubation at 37 °C with 5% CO_2_. After 24 h, the medium was replaced with osteogenic differentiation medium, which comprised a growth medium supplemented with 50 µg/mL ascorbic acid (Sigma-Aldrich, Edinburgh, UK), 10 mM β-glycerophosphate (Sigma-Aldrich, Edinburgh, UK), dexamethasone 10-7M (Sigma-Aldrich, UK), and 0.01 mL of TCP and 0.3B-TCP solutions, obtained as described above.

aMSCs cultured in growth medium served only as the negative control, while those cultured in osteogenic differentiation medium acted as the positive control for the experiment. Each experiment was repeated in triplicate, and cells were incubated at 37 °C with 5% CO_2_, with medium changes every two to three days for a duration of 3 weeks. Red calcified deposits were visualized by staining with Alizarin Red S (Sigma-Aldrich, Edinburgh, UK), highlighted in red. Cell images were captured using an inverted optical microscope (Nikon, Eclipse T2000-U, Hamburg, Germany).

### 2.7. Statistical Analysis

The antimicrobial activity tests and MTT tests were performed in triplicate. The mean OD of 600 nm ± standard deviation (SD) was analyzed using the non-parametric Dunnett test for multiple comparisons (SAS JMP Statistical Discovery software v14 pro, Milan, Italy). *p* values of ≤0.05, ≤0.01, and ≤0.001 were considered statistically significant, as indicated in the figure legends.

## 3. Results and Discussion

**Figure 1:**
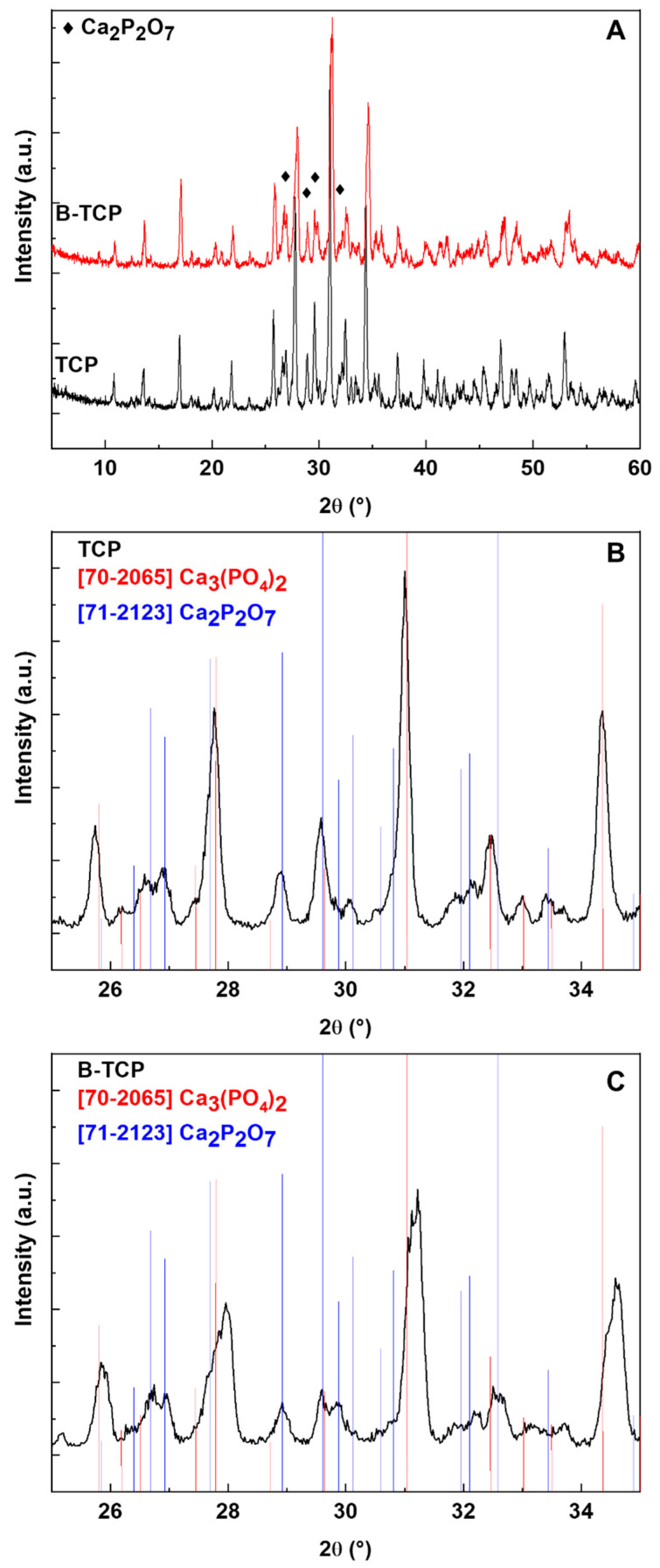
X-ray diffraction patterns of TCP and B-TCP ceramics. (**A**)—general pattern; (**B**)—interval 2θ 25–35° for β-TCP; (**C**)—interval 2θ 25–35° for B- β-TCP.

According to the obtained XRD results, the main phase of the ceramics is β-TCP, while the second phase is Ca_2_P_2_O_7_ (see Figure 1A), with an estimated content of 30 wt.%, by means of the corundum numbers method [33,34]. As seen in Figure 1B, there is some shift towards higher angles for the diffraction peaks of β-TCP compared to the ICDD card data 70-2065, whereas for the impurity phase Ca_2_P_2_O_7_, the diffraction peaks show almost no shift relative to the ICDD card data 71-2123 (Figure 1B,C). This suggests that the boron present in the sample enters specifically into the β-TCP phase, likely (considering the data [27]) in the form of BO_3_^3−^ ions, replacing PO_4_^3−^ ions.

In our study, as demonstrated by X-ray diffraction data, a mixture of whitlockite and calcium pyrophosphate (CPP) phases was formed. The parameters of the whitlockite crystal lattice changed significantly, while the parameters of the CPP lattice remained practically unchanged, confirming that boron enters the whitlockite lattice, most likely in the form of the BO_3_^3−^ ion, which replaces the phosphate ion PO_4_^3−^.

Based on the elemental analysis data obtained using inductively coupled plasma mass spectrometry, it was established that the P/B ratio in the synthesized borophosphate was 5.7, correlating with the ratio introduced during synthesis.

As shown earlier in the literature, in the structure of tricalcium phosphate, it is possible to substitute Ca^2+^ cations with transition metal cations [35] and phosphate PO_4_^3−^ anions for other groups, such as borate BO_3_^3−^ or others [23]. Due to the difference in ionic radii, distortion of the whitlockite structure may occur, accompanied by partial destruction of the latter and, consequently, the appearance of a secondary phase.

**Figure 2:**
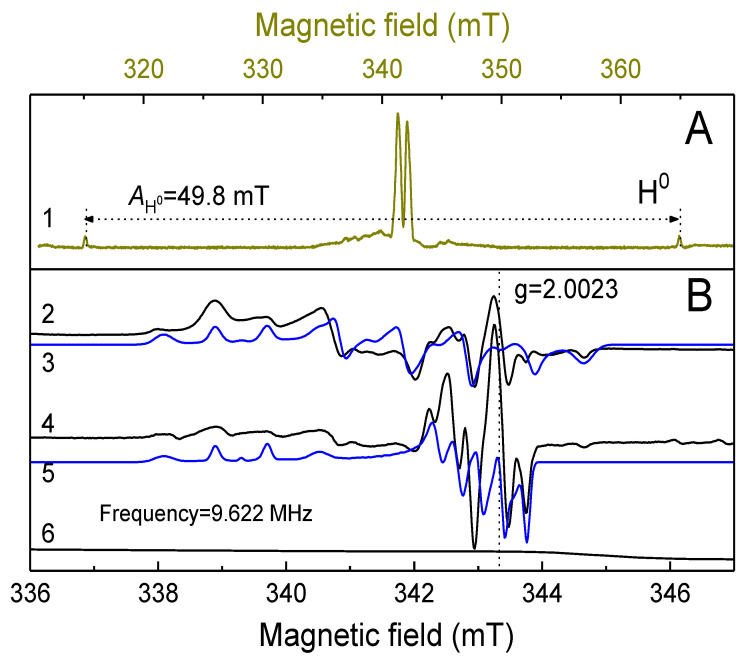
(**A**) pulse EPR and (**B**) CW EPR spectra of TCP samples. Arabic numbers indicate: 1 pulse EPR spectrum recorded by the method of measuring the electron spin echo (pulse EPR). 2 EPR spectrum registered at 2 mW. 3 (blue curve) simulation of the EPR spectrum of the 1st center (PC1). 4 (black curve) EPR spectrum registered at 0.02 mW. 5 (blue curve) simulation of the EPR spectrum with two paramagnetic centers (PC1 and PC2). 6 (black curve) EPR spectrum before X-ray irradiation of the sample.

The results of the EPR studies on B-TCP samples under various experimental conditions are presented in Figure 2. In the non-irradiated samples, a broad, low-intensity line is observed (Figure 2B, spectrum 6). Following X-ray irradiation, an intense spectrum emerges, exhibiting a shape characteristic of paramagnetic centers in powders. By changing the EPR registration conditions, we were able to isolate at least some of these centers to determine their spectroscopic parameters and origins.

In the spectra recorded in the pulse mode (spectrum 1, Figure 2A), by measuring the intensity of the electron spin echo, as well as in CW mode it is possible to detect (isolate from other types of paramagnetic centers) trapped atomic hydrogen centers H^0^ (doublet due to the nuclear magnetic moment of proton I = ½, therefore 2I + 1 = 2, with the characteristic splitting of about 49.8 mT) [36]. Considering the XRD results (Figure 1), the results of our previous studies of TCP [30], and recent EPR investigations of various calcium pyrophosphate polymorphs [36], we suggest that the observed hydrogen is trapped in the impurity phase Ca_2_P_2_O_7_. Unfortunately, the short transverse electronic relaxation time of the paramagnetic centers observed in pulse mode (T_2e_ = 2 ± 0.5 microseconds at T = 300 K) prevents the identification of other paramagnetic centers (the lines in the vicinity of the magnetic fields of 336–348 mT) using the pulse mode of the spectrometer in the same manner as that shown in ref. [30].

Analysis of the CW EPR spectra (Figure 2B), detected at different values of microwave power, 2 mW (line 2) and 0.02 mW (line 4), allows for the separation of other types of paramagnetic centers (PC) with the spectroscopic parameters presented in Table 1. At least two more anisotropic components of the EPR spectrum from two different centers (referred to below as PC 1 and PC 2, respectively) can be distinguished.

At 2 mW, only PC 1 is observable, while at lower microwave power, the total spectrum (a superposition of the two) is detected. For each component, groups of 4 lines equidistant from each other can be distinguished in the EPR spectra, suggesting that they are due to the hyperfine interaction with the nuclear magnetic moment of the most common boron isotope, ^11^B, with a nuclear spin I = 3/2 (2I + 1 = 4). We have selected spectrum simulations (shown by blue curves 3 and 5 in Figure 2B) for these components, with the approximation parameters shown in Table 1. Since β-TCP has low rhombohedral R3c symmetry, each spectrum was simulated with three components for the spectroscopic g-factor and hyperfine (A) tensors.

The obtained values of the components of g-factors and hyperfine interaction components A, ranging from 23–28 MHz for PC 1, are in good agreement with the known literature data for the boron radical anion BO_3_^2−^ [37,38]. For the boron radical cation, the value reported in the literature is much less, i.e., A_iso_ = 3.3 MHz [39]. The EPR parameters for PC 2 differ significantly from the literature data and, therefore, the origin of PC 2 cannot be unambiguously defined from our experiments. As was shown recently in ref. [36], while EPRs of the radiation-induced paramagnetic centers are extensively studied in some calcium phosphates (such as TCP or hydroxyapatite), there are still many undefined paramagnetic centers, even in the nominally pure pyrophosphate polymorphs. The obtained PC 2 center is probably due to the boron-containing radicals in the impurity phase.

**Figure 3:**
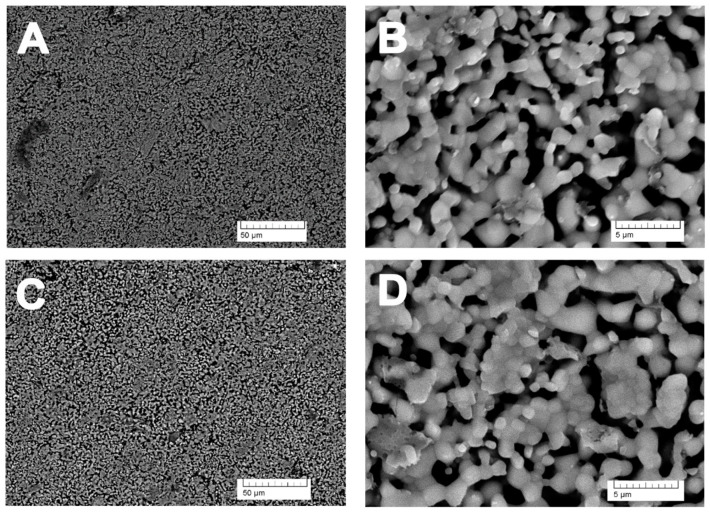
SEM image of ceramics from 0.3 B-TCP at different magnifications: (**A**,**B**) before soaking, (**C**,**D**) after soaking.

The microstructure of ceramics obtained from borate-containing TCP sintered at 1100 °C (Figure 3A,B) is homogeneous. According to SEM data, the grain size of ceramics is 1–2 µm and the pore size is 2–5 µm.

**Figure 4:**
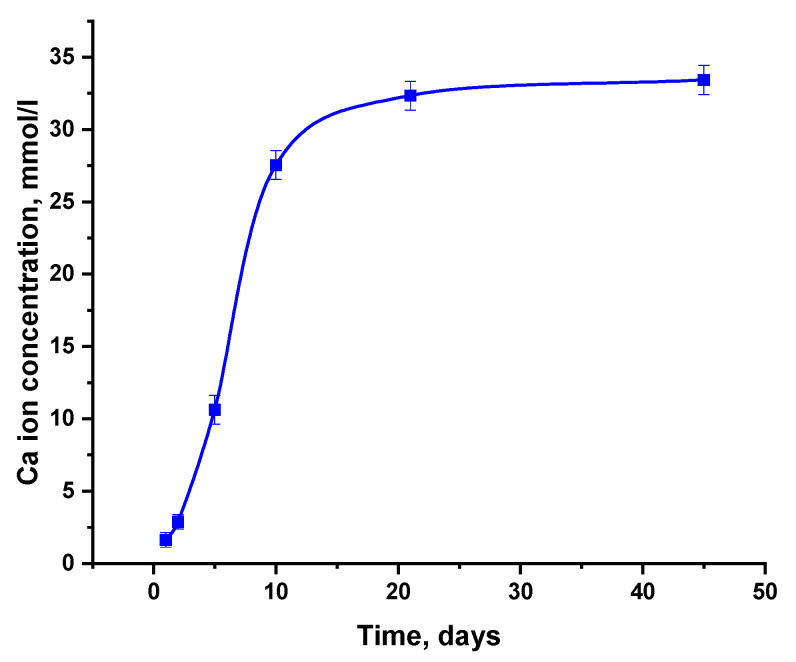
Time dependence of calcium ion concentration in saline solution upon soaking B-TCP ceramics.

The B-TCP ceramic was soaked in a saline solution with TRIS buffer at pH 7.4 and 37 °C. The experiment involved immersing the samples of B-TCP ceramics in the solution for 1, 2, 5, 10, 20, and 45 days. After each specified time, the calcium content in the solution was measured, and the solubility curve is presented in Figure 4. As can be observed, at the first stage of dissolution, the concentration of calcium ions increased due to the solubility process; then, after 30 days, the curve reached a plateau, which indicated the establishment of a dynamic equilibrium between calcium ions transferred to physiological solution as a result of ceramics dissolution and calcium ions removed from solution due to the formation of a layer of biological apatite on the surface of ceramics, according to the reaction (3):

(10-d)Ca^2+^ + (6-x-y)PO_4_^3−^ + zOH^−^ + yCO_3_^2−^ + nH_2_O → Ca_10-d_(HPO_4_)_x_(PO_4_)_6-x-y_(CO_3_)_y_(OH)_z_ · nH_2_O (3)

The microstructure of the ceramics after being kept in the saline solution for 45 days did not visibly change (Figure 3C,D).

**Figure 5:**
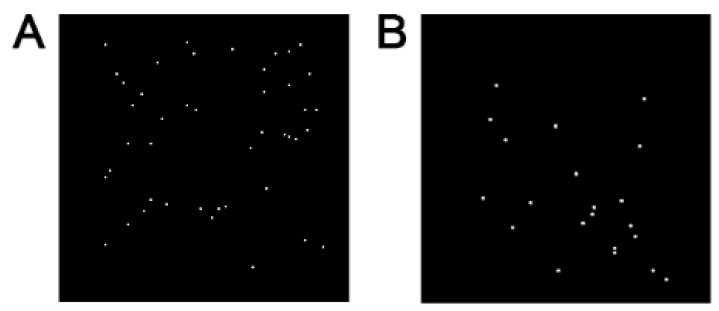
Boron distribution in B-TCP ceramics obtained by the SEM-EDX method: (**A**)—before soaking; (**B**)—after soaking.

Boron was homogeneously distributed in the B-TCP ceramic (Figure 5A). After soaking the ceramics in saline solution for 45 days, a part of the boron atoms remained within the ceramics (Figure 5B).

The porosity of the ceramics, measured by hydrostatic weighing, is approximately 32.5 ± 1%, aligning with the porosity levels observed in ceramics obtained through the method described in [40]. The pore size varied from several tens of nm to tens of microns.

**Figure 6:**
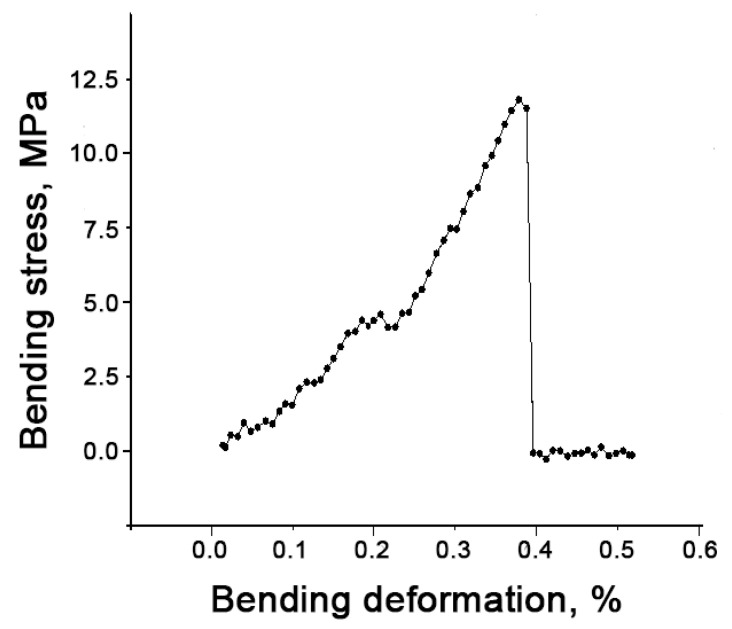
Loading curve of B-TCP ceramic.

The bending strength of B-TCP ceramic was determined to be 30 ± 3 MPa (Figure 6), which is comparable to the strength of β-TCP ceramic (35 ± 3 MPa), as reported in [40]. The low strength of the ceramics can be attributed to their relatively high porosity. The loading curve shown in Figure 6 exhibits a characteristic form indicative of brittle fracture, characteristic of ceramic materials [41].

The outcomes of microorganism growth (*S. aureus*, *E. coli*, *P. aeruginosa*, *E. faecalis*, and *C. albicans*) in the presence of TCP and 0.3 B-TCP are outlined below. The growth of each microorganism was assessed after 24 h of incubation at its respective optimal growth temperature.

**Figure 7:**
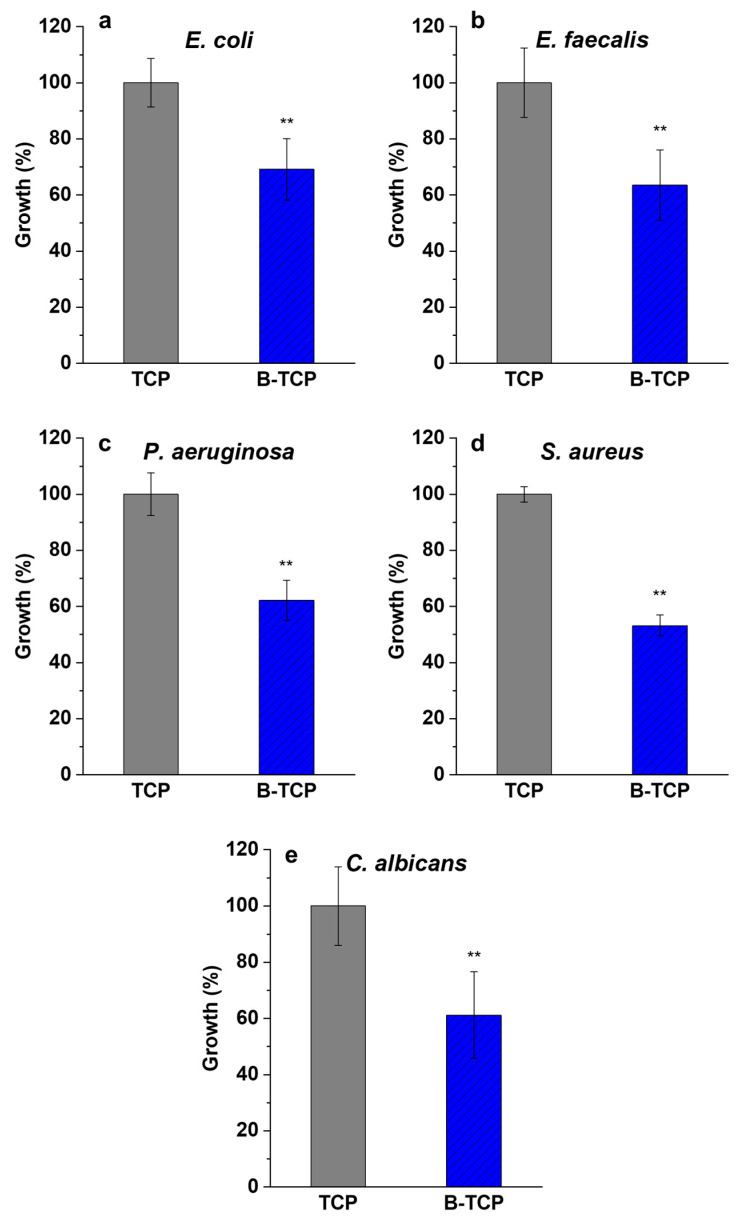
Growth (%) and standard deviation (SD) for (**a**) *E. coli*, (**b**) *E. faecalis*, (**c**) *P. aeruginosa*, (**d**) *S. aureus*, and (**e**) *C. albicans* cultured in the presence of TCP and B-TCP. The reported values were obtained from three independent experiments and expressed as mean percentage values ± SD, compared to growth in the presence of TCP, considered as a control set at 100%. *p* values (Dunnett test): *p* < 0.01 ** compared to positive control.

In all tests shown in Figure 7, statistically significant inhibitory effects on microbial growth were observed in the presence of 0.3 B-TCP compared to growth in the presence of TCP, considered as a control set at 100%. Specifically, with 0.3 B-TCP, the growth inhibition rates were as follows: (a) 30.9% for *E. coli*, (b) 36.4% for *E. faecalis*, (c) 37.8% for *P. aeruginosa*, (d) 46.8% for *S. aureus*, and (e) 38.8% for *C. albicans*.

From the results of the antimicrobial test, which involved four bacteria (two Gram-negative and two Gram-positive) and the fungus, there was no evidence of stronger growth inhibition of one type of bacteria (Gram-positive or Gram-negative), nor was there evidence of specific inhibition of the fungus compared to the bacteria.

**Figure 8:**
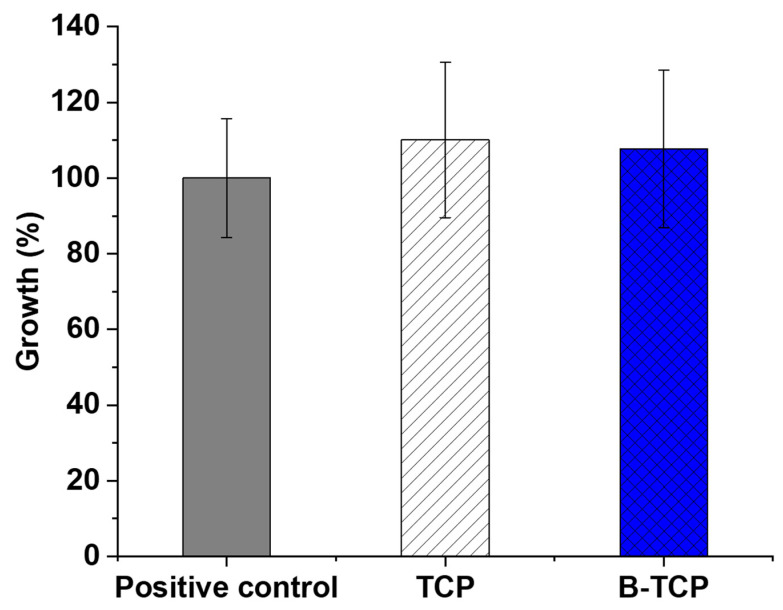
Assessment of growth (%) for aMSCs under different conditions: alone, in the presence of TCP, and B-TCP at 24 h, incubated at 37 °C, using MTT assay. The reported values were obtained from three independent experiments and expressed as mean percentage values ± SD compared to the values of the positive control corresponding to 100%.

The MTT test enables the assessment of the prepared ceramics’ toxicity on cells. In this study, the examination involved TCP and 0.3B-TCP compounds with third-passage aMSCs. Figure 8 illustrates the growth percentage alongside the standard deviation derived from the experiment’s average OD600 nm values.

Data analysis revealed that the growth of aMSCs in the presence of TCP was 110.1%, while the cell growth in the presence of 0.3 B-TCP was 107.7% compared to the positive control. In both experimental conditions, no toxicity was observed, as aMSCs growth did not show statistically significant differences in the presence of TCP and 0.3 B-TCP compared to the control.

**Figure 9:**
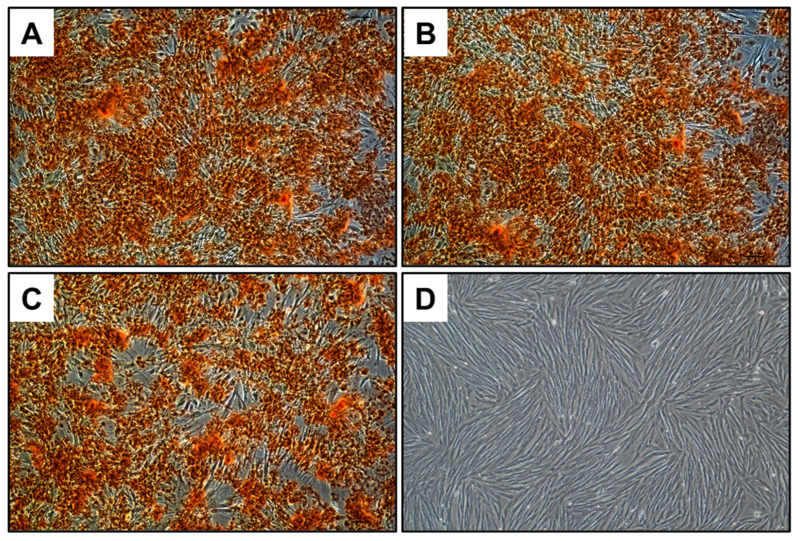
Osteogenic differentiation of aMSCs in the presence of (**A**) TCP, (**B**) 0.3 B-TCP, and in the absence of (**C**) positive control and (**D**) negative control. The images were registered at 10× magnification.

The osteogenic differentiation potential of the aMSCs was assessed in the presence of both TCP and 0.3 B-TCP. Calcium deposits in the extracellular matrix were visualized in red through Alizarin Red S staining. As shown in Figure 9, the aMSCs demonstrated comparable osteogenic differentiation potential in the presence of both TCP and 0.3 B-TCP, with no significant differences observed. In all experimental conditions, aMSCs differentiated into the osteogenic lineage, similarly to the positive control.

To summarize, the investigation of the cytocompatibility of TCP and 0.3 B-TCP yielded excellent results, as demonstrated by both MTT tests and the differentiation into the osteogenic lineage of aMSCs. The MTT assay revealed statistically comparable cell growth and viability compared to the positive control for both TCP and 0.3B-TCP. Furthermore, both tests underscored the cytocompatibility of TCP and 0.3 B-TCP, showing no adverse effects on the osteogenic differentiation of aMSCs. These favorable characteristics position 0.3B-TCP ceramic as potential candidates for future applications in regenerative medicine.

## 4. Conclusions

XRD analysis revealed that the synthesis of 0.3B-TCP performed using boric acid as precursor, followed by heat treatment at 900 °C, resulted in the formation of a boron compound with a whitlockite structure. Calcium pyrophosphate, 30 wt.%, was detected by XRD as an impurity phase. EPR spectroscopy demonstrated the replacement of PO_4_^3−^ phosphate anions in the β-TCP structure by BO_3_^3−^ borate anions.

Porous 0.3B-TCP ceramic was successfully obtained through the method of two-sided uniaxial pressing followed by sintering at 1100 °C. The measured porosity of the ceramics was 32.5 ± 1%, aligning with the porosity level achieved using the same method.

The bending strength of the B-TCP ceramic was found to be 30 ± 3 MPa, which is comparable to previously obtained results for ceramics made of β-TCP (35 MPa).

Investigation of the behavior of B-TCP ceramics in the saline solution with TRIS buffer revealed that after 30 days, the concentration of calcium ions reached a plateau. This phenomenon can be explained by the dynamic equilibrium between calcium ions in solution and those forming a layer of biological apatite on the surface. Boron remained present within the ceramic matrix after 45 days of immersion of the ceramic in the saline solution.

For B-TCP, a significant inhibitory effect was observed for all tested microorganisms. Specifically, the inhibition was 30.9% for *E. coli*, 36.4% for *E. faecalis*, 37.8% for *P. aeruginosa*, 46.8% for *S. aureus,* and 38.8% for *C. albicans*, compared to pure TCP.

The evaluation of the cytocompatibility of TCP and 0.3B-TCP through the MTT assay revealed positive results, with the growth and viability of aMSCs statistically comparable to the positive control for both TCP and 0.3B-TCP. Additionally, aMSCs exhibited osteogenic differentiation potential comparable to the positive control for both ceramic materials.

The combination of antibacterial efficacy and good cytocompatible characteristics renders 0.3B-TCP a prospective bone substitute ceramic material.

## Figures and Tables

**Table 1:** Spectroscopic g-factor and hyperfine (A) tensors used to simulate the EPR spectra detected in B-TCP with two boron-containing paramagnetic centers (PC).

	*g*_x_	*g*_y_	*g*_z_	A_x_ (MHZ)	A_y_ (MHZ)	A_z_ (MHZ)
PC 1	2.0042(2)	2.010(2)	2.028(2)	25(1)	28(1)	23(1)
	2.0039(2)	2.0054(2)	2.018(2)	8(1)	10(1)	23(1)

## Data Availability

The data are available upon a reasonable official request to the corresponding author.
